# Differences in Cytokine Expression and STAT3 Activation between Healthy Controls and Patients of Unexplained Recurrent Spontaneous Abortion (URSA) during Early Pregnancy

**DOI:** 10.1371/journal.pone.0163252

**Published:** 2016-09-22

**Authors:** JunYing Cai, MuJun Li, QianYi Huang, XiaoQian Fu, HuiMei Wu

**Affiliations:** Department of Reproductive Center, The First Affiliated Hospital of Guangxi Medical University, Nanning, Guangxi, China; Xavier Bichat Medical School, INSERM-CNRS—Université Paris Diderot, FRANCE

## Abstract

Unexplained recurrent spontaneous abortion (URSA) is a common complication of pregnancy. Although tolerance of the maternal immune system is considered to be essential for a normal pregnancy, the precise mechanism underlying the pathogenesis of URSA remains to be fully elucidated, albeit it is known to involve inflammation. Here, we examine the relationship between the expression of inflammatory cytokines and the activation of downstream signaling pathways in URSA patients. Decidual and peripheral blood samples were collected from 30 URSA patients and from 30 women with normal early pregnancies. Western blot analysis was used to measure the expression levels of signal transducers and activators of transcription 3(STAT3), phosphorylated STAT3(p-STAT3), and interleukin-17 receptor(IL-17R) in the decidual samples. Enzyme-linked immunosorbent assay was used to assess the levels of IL-17, IL-10, IL-6, and IL-23 in the peripheral blood and decidual samples. In the URSA patients, the IL-10 expression levels were lower than those in the control subjects (*P*<0.05), whereas IL-6, IL-17, and IL-23 were all expressed at higher levels(*P*<0.05). Furthermore, the expression levels of IL-17R and p-STAT3 were higher in the URSA patients, exhibiting a trend similar to that of IL-23. Our finding of increased IL-23 expression in the deciduae and peripheral blood of patients with URSA suggest that this maybe a contributing factor to the pathogenesis of this disease. Likewise, STAT3 activation through its phosphorylation, which was associated with the IL-23 increase, may also be involved in URSA pathogenesis. However, the precise pathogenic mechanism requires further study.

## Introduction

Recurrent miscarriage (RM), which occurs in 1%–5% of pregnancies [1], is defined as the occurrence of two or more pregnancy losses before the 20th week of gestation. RM is caused by several factors, including chromosomal, anatomic, endocrinologic, infectious, and autoimmunologic abnormalities. In approximately 50% of cases, however, the precise cause remains unknown. Such cases, which occur primarily in the first trimester [2], are referred to as unexplained recurrent spontaneous abortion (URSA). In recent years, multiple studies have found that immune imbalance at the maternal-fetal interface plays a role in the pathogenesis of URSA, and interactions among an array of cytokines are believed to contribute to the ability of the maternal immune system to tolerate the genetically incompatible fetus. Decidual tissue, an important component of the maternal-fetal interface, contains decidual stromal cells and decidual immune cells, including T cells, uterine natural killer cells, and macrophages [3]. Decidual cells, which are developed from the endometrium, are regulated by ovarian steroid hormones after blastocyst implantation. Decidual tissue is essential for germ cell implantation and embryo development organization. Furthermore, it has the functions of nourishing the blastocysts, adjusting the endocrine milieu, regulating trophocyte invasion, and protecting the embryo from maternal rejection, thus playing an important role in normal pregnancy. Therefore, changes in the pattern of cytokine expression may result in inflammation of the endometrial microenvironment, leading to spontaneous abortion [2, 4, 5]. As is known, T helper 17 (Th17) and T regulatory(Treg) cells are subsets of CD4+T cells. Many studies have reported that the Th17/Treg paradigm plays a key role in the establishment and maintenance of maternal-fetal immune tolerance [6, 7].

Treg cells are essential for maintaining immunologic unresponsiveness to self-antigens and for suppressing excessive immune responses that are deleterious to the host [8]. In particular, Treg cells secrete the anti-inflammatory cytokine interleukin-10(IL-10), which can inhibit the secretion of a variety of inflammatory cytokines and inhibit the activation of Th1 and Th17 cells [9]. Deficits in the number and/or function of Treg cells have been documented in cases of abortion in URSA [10, 11]. Conversely, Th17 cells are characterized by their production of potent pro-inflammatory cytokines, such as interleukin (IL)-17, which plays a major role in rejecting conceptus antigens and may therefore be harmful to the maintenance of pregnancy [12]. In a previous study of spontaneous abortion cases, Nakashima *et al*. [13] detected the accumulation of IL-17^+^ cells in the decidua, which was also appositively correlated with the number of neutrophils present in the tissue [13]. Notably, IL-23 contributes to the maintenance and expansion of Th17 cells and stimulates the production of pro-inflammatory effector cytokines such as IL-17[14, 15]. When bound to its receptor IL-17R, IL-17 induces the release of cytokines that promote inflammatory processes and can lead to spontaneous abortion [16].

Signal transducers and activators of transcription 3(STAT3) is a transcription factor that plays critical roles in the development, cell growth, and homeostasis of a variety of tissues [17], and has also been reported to contribute to the pathogenesis of autoimmunity [18, 19]. STAT3 activation is mediated by the binding of IL-6 to its receptor, and the activated STAT3 in turn promotes upregulation of IL-6 expression via a positive feedback loop [20]. A successful pregnancy is dependent on maternal immunologic tolerance to the paternal antigen of the embryo, and STAT3-dependent signals are essential for the differentiation and/or expansion of human IL-17-producing T cell populations. Although STAT3 expression has been detected in deciduae, and its variants are associated with RM [21], the exact mechanism by which it contributes to URSA is unclear.

In the present study, to improve our understanding of the pathogenesis of URSA, we assessed the cytokine balance/imbalance at the maternal-fetal interface by measuring the expression levels of IL-6, IL-10, IL-17, and IL-23, as well as the protein expression levels of IL-17R, STAT3, and phosphorylated STAT3 (p-STAT3), in the peripheral blood and/or decidual tissues of URSA patients and of women with normal early pregnancies. The results of this study revealed that there is a similar trend of increased IL-23 and p-STAT3 expression in URSA patients, indicating that these molecules may contribute to the pathogenesis of URSA and may therefore serve as potential therapeutic targets for its prevention.

## Materials and Methods

### Study design

This study was approved by the ethics committee of the First Affiliated Hospital of Guangxi Medical University (Nanning, China). Prior to artificial miscarriage, the patients and controls were informed that their decidual tissues would be collected and frozen for research purposes, and informed written consent was obtained from each participant. All study participants were patients of the outpatient department of the Gynaecology Clinic at the First Affiliated Hospital of Guangxi Medical University between January 2015 and October 2015. All decidual and peripheral blood samples were collected from 30 URSA patients as well as 30 women with normal early pregnancies. All participants were early pregnant women, and the decidual tissues of the two groups were obtained by artificial miscarriage rather than medical miscarriage. The decidual and villi tissues were obtained through negative-pressure aspiration, and based on the characteristic morphology of the decidua and villi, the decidual tissue was identified and isolated into freezing tubes. The tissue samples were transported in liquid nitrogen to the laboratory and stored at -80°C until experimental use. For analysis of the peripheral blood, 5mL of blood was collected from the antecubital vein of each study participant into serum separator tubes.

The URSA group was defined as women who had experienced two or more consecutive abortions before week 12 of gestation. The diagnosis of URSA was made after excluding any verifiable causes, such as abnormalities of the uterus or cervix, chromosomal abnormalities, infection, endocrine and metabolic diseases, congenital thrombophilias, and autoimmune diseases [10]. The average number of abortions in the URSA group was 2.40±0.59. The male partner of each woman in the study had a normal semen status, according to criteria of the World Health Organization (2011). All patients were diagnosed with intrauterine pregnancy by ultrasound (GE LOGIC-E9, USA) at 7 and 9 weeks of gestation. For control subjects, we randomly selected 30 healthy women in early pregnancy who were undergoing artificial miscarriage (selective abortion). Fetal heart activities were identified within 1 week prior to the miscarriage. According to the diagnostic criteria for URSA, in the event of no normal fetal heart beat, the patients were recommended to undergo an ultrasound examination as well analysis of their serum beta human chorionic gonadotropin (β-HCG) levels, the latter to be repeated after a week. If the β-HCG levels had decreased and the fetal heart beat was no longer detectable, the patients were suggested to undergo artificial miscarriage. URSA patients underwent early miscarriages at an average of 50.90 ± 5.20 days of gestation, and the mean maternal age was 28.25 ± 3.13 years (range: 24–33 years). Meanwhile, the mean gestational age of the controls at termination of pregnancy was 51.80 ± 5.03 days and the mean maternal age was 27.05 ± 2.80 years (range: 23–33 years). There were no significant differences in the mean age and gestational age between the two groups.

### Enzyme-linked immunosorbent assay

The IL-17, IL-10, IL-6, and IL-23 levels in the decidual tissue and blood serum samples of all the study participants were measured by enzyme-linked immunosorbent assay(ELISA). Each sample (300 mg) of decidual tissue was cut into 1mm^3^ pieces, homogenized, and centrifuged, and the resulting tissue suspensions were stored at -80°C until analysis. For the peripheral blood, each 5mL sample was allowed to clot for 30min and then centrifuged, after which the upper serum layer was collected and stored at -80°C until use. All samples were thawed only once. The ELISA was carried out using commercial kits in accordance with the manufacturer’s instructions (Shanghai Yuanye Biotechnology Co., Ltd., Shanghai, China). All experiments were performed in duplicates. The sensitivity of the ELISA kit was >1 pg/mL for IL-6 and IL-23, and >0.1 pg/mL for IL-10 and IL-17.

### Western blotting

Western blot analysis was used to measure the STAT3, p-STAT3, and IL-17R expression levels in the decidual tissue samples of the study participants. Total protein was extracted from 300 mg of each decidual sample, and the resulting protein concentrations were measured using a nucleic acid protein detector (ThermoScientific, Waltham, MA, USA). All the samples were adjusted to a protein concentration of 20μg/mL by using distilled water. Approximately 80μL of each protein sample was mixed with 20uL of 5×sodium dodecyl sulfate-polyacrylamide gel electrophoresis(SDS-PAGE) loading buffer (Baomanbio, Shanghai, China), boiled for 5min, and allowed to cool for 5min at room temperature. For western blot assay, 100μg of each protein sample was separated by 10% SDS-PAGE and then transferred to polyvinylidene difluoride membranes (0.45μm; Millipore Corp., Billerica, MA, USA). The membranes were blocked by incubation with 0.5% bovine serum albumin (diluted in Tris-buffered saline) for 2h and then incubated with antibodies specific to STAT3[1: 2, 000 dilution; Cell Signaling Technology (CST), Danvers, MA, USA], p-STAT3 (1:2, 000; CST), IL-17R (1:1, 000; Novus, St. Louis, MO, USA), and glyceraldehyde-3-phosphate dehydrogenase (GAPDH; 1: 5, 000; Vazyme Biotech, Nanjing, China), according to the manufacturers’ instructions. Subsequently, the membranes were incubated for 1h with a fluorophore-labeled secondary antibody (1: 10,000; LI-COR Biosciences, Lincoln, NE, USA) diluted in secondary antibody dilution buffer, and the labeled proteins were then detected using a sweep membrane apparatus(LI-COR). Staining of GAPDH was used to assess the loading and transfer quality. Relative protein expression levels were analyzed using Image J software (National Institutes of Health, Bethesda, MD, USA).

### Statistical analyses

Data are presented as the means ± standard deviations(SDs). Statistical analyses were performed using SPSS statistical software version 16.0(SPSS Statistics, Inc., Chicago, IL, USA). Data were tested with the K-S inspector; the data conformed to normal distributions. Differences between groups were assessed using Student’s *t*-tests, and *P*<0.05 was considered statistically significant.Error bars in figures denote the SDs.

## Results

### Comparison of the IL-6, IL-10, IL-17, and IL-23 levels between the two groups

**Fig 1:**
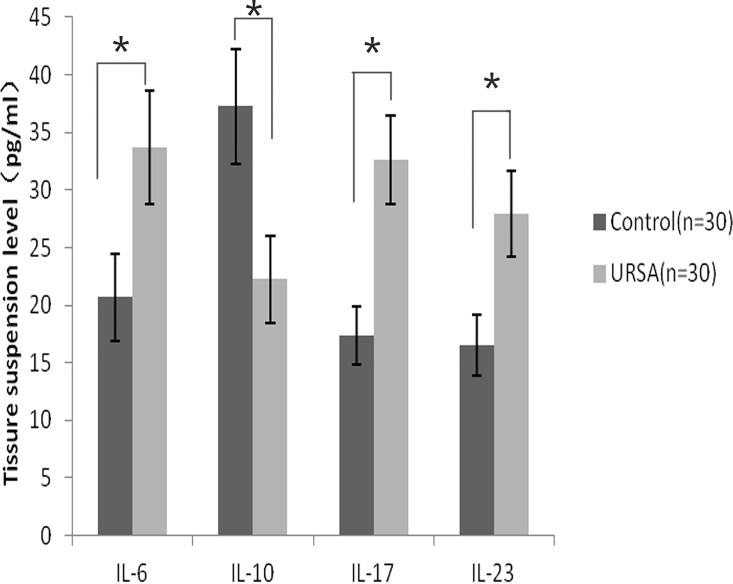
Comparison of cytokine expression profiles in decidual tissue samples from patients in the unexplained recurrent spontaneous abortion(URSA) group and the control group. The enzyme-linked immunosorbent assay was used for the analysis. Error bars represent standard deviations. *P<0.05.

**Fig 2:**
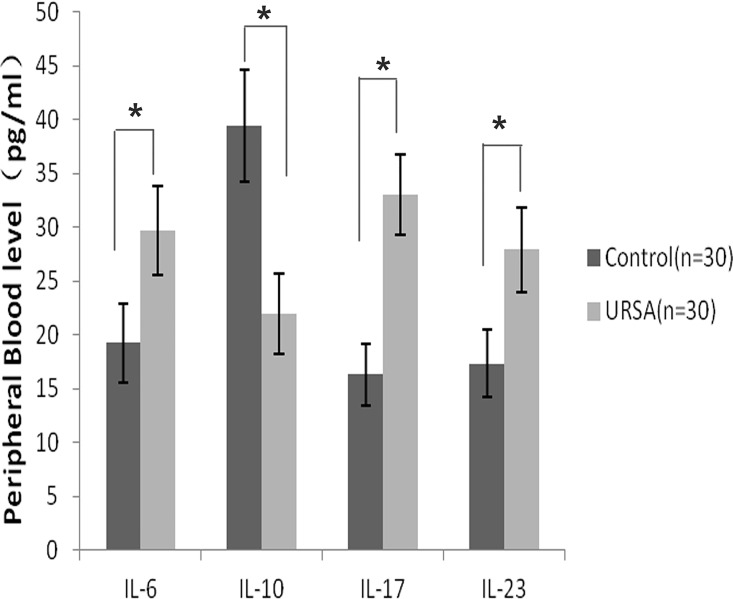
Comparison of cytokine expression profiles in peripheral blood samples from patients in the unexplained recurrent spontaneous abortion(URSA) group and the control group. The enzyme-linked immunosorbent assay was used for the analysis. Error bars represent standard deviations. *P<0.05.

ELISA analysis of the tested interleukin levels in the human decidual tissues showed that IL-10 was expressed at lower levels in the URSA group (Fig 1). In contrast, the levels of IL-6, IL-17, and IL-23 were significantly higher in the URSA group than in the control tissues(*P*<0.05). Similar results were obtained from the ELISA analysis of the serum samples from the two groups (Fig 2).

### Comparison of the STAT3, p-STAT3, and IL-17R levels in both groups

**Fig 3:**
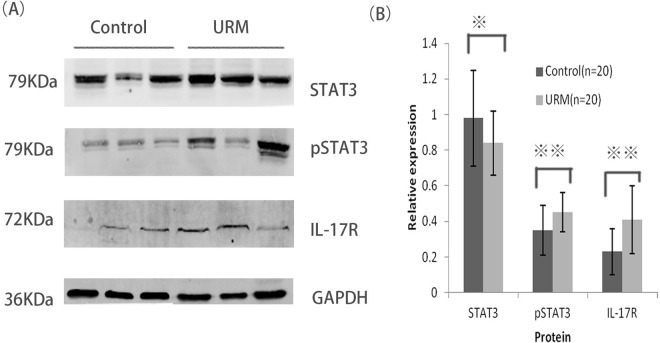
Comparison of signal transducers and activators of transcription 3 (STAT3), phosphorylated STAT(p-STAT3), interleukin-17 receptor(IL-17R), and glyceraldehyde-3-phosphate dehydrogenase (GAPDH) protein expression levels in decidual tissue harvested from women in the control and unexplained recurrent spontaneous abortion(URSA) groups. **(A)** Image depicting the expression level of each indicated protein in the control (n = 30) and URSA(n = 30) groups, as detected by western blot analysis. GAPDH was used as a loading control to normalize the protein levels. (**B)** The relative expression level of each protein was calculated from its densitometric intensity using Image J software. Comparison the respective proteins levels between the two groups are included. Error bars indicate standard deviations. *P>0.05; **P<0.05.

Increased p-STAT3 and IL-17R levels were detected in the decidual tissues of patients with URSA, compared with those in the control group (*P<* 0.05; Fig 3); however, there was no significant difference in the STAT3 expression levels between the two groups(*P*>0.05; Fig 3). Notably, in the decidual samples, there was a corresponding trend between the levels of IL-17 secretion and the expression levels of IL-23 and p-STAT3. These findings indicate that the pro-inflammatory function of IL-23 and IL-17 may be closely linked to the phosphorylation of STAT3.

## Discussion

Embryos express paternal antigens that are foreign to the mother. As such, these antigens could be viewed as being analogous to an allograft. Therefore, the induction of maternal immunosuppression of the embryo/fetus is a main concern for maintaining maternal-fetal tolerance. Indeed, immune dysregulation of the maternal-fetal interface plays a critical role in the pathogenesis of URSA. Previous studies [22] of successful pregnancies have detected a predominance of Th2 over Th1 cytokines, and of Treg over Th17 cytokines, at the maternal-fetal interface. Early research has demonstrated that IL-23 plays a crucial role in certain Th1-mediated autoimmune diseases, such as in experimental models for allergic encephalomyelitis, arthritis, and chronic bowel inflammation [23]. Although various studies of URSA have been conducted, the mechanism responsible for URSA remains unclear. To the best of our knowledge, this is the first analysis of the pathogenic mechanism of URSA from the perspective of the expression of URSA related cytokines and the STAT3 signaling pathway. Specifically, we evaluated the protein levels of p-STAT3, IL-17R, and STAT3, as well as the expression levels of IL-6, IL-17, IL-10, and IL-23 in decidual tissues harvested from URSA and control patients. We detected increased expression of IL-6, IL-17, IL-23, p-STAT3, and IL-17R, and decreased expression of IL-10 in the deciduae and peripheral blood of the URSA group relative to the control subjects. Notably, the elevated expression of IL-23 and IL-17, which could be directly responsible for the observed increase in p-STAT3, is consistent with previous findings reported by Saifi *et al*. [7]. Furthermore, our findings are in agreement with previous data showing the overexpression of Th17 signature cytokine genes in recurrent spontaneous abortions [10].

IL-17 is a potent inducer of inflammation that functions to promote the cellular infiltration and production of several pro-inflammatory cytokines and chemokines [24], including IL-6, which is involved in eliciting Th17 cytokine production and subsequent embryo wastage. Meanwhile, IL-23, which is expressed in macrophages and dendritic cells, can help to maintain or stabilize the Th17 response. IL-23R is found on memory T cells, natural killer T cells, macrophages, dendritic cells, and naive T cells upon activation by transforming growth factor-beta(TGF-β) and IL-6[25]. After IL-23 binds to its specific receptor, STAT3 is immediately phosphorylated, resulting in its dimerization and migration to the nucleus, thereby inducingthe expression of molecules that play roles in a variety of functions [16]. Patients with ulcerative colitis exhibited persistently elevated expression of total STAT3 and p-STAT3, a phenomenon that was positively correlated with the degree of inflammation [26]. At the maternal-fetal interface, the balance of local pro-inflammatory and anti-inflammatory cytokines is important for a successful pregnancy. IL-10 is a Th2 cytokine and is known to selectively suppress Th1-mediated cellular responses by inhibiting the production of inflammatory cytokines [27], and to mediate the inhibitory effects of Treg cells. Specifically, IL-10 exerts its signaling effects through the IL-10 receptor, resulting in inhibition of Th17 cytokines and retinoic acid-related orphan receptor gammaT(RORγt) protein expression, thereby decreasing the amount of IL-17 produced and preventing exaggerated inflammatory and immune responses [28]. In this study, we found that IL-10 expression was decreased in the peripheral blood and decidual tissue of URSA patients, which is consistent with the results obtained by Piccinni *et al*.[29]. Meanwhile, the increased levels of IL-6 in the decidua and peripheral blood, as well as increased p-STAT3 expression in the decidua of the URSA patients in this study are in accordance with a previous report [30], in which IL-6 was found to be the main activator of STAT3, and the IL-6-dependent STAT3 activation was suggested to be involved in IL-23R upregulation.

Several studies have shown that STAT3 is activated in response to a wide variety of cytokines, including IL-6, IL-21, and IL-23[31, 32]. We found that the increases in the levels of p-STAT3 in the URSA group were accompanied by increases in the levels of IL-23 and IL-6. Since STAT3 physically associates with IL-23R [25], we conclude that the increased IL-23 expression observed in the decidual tissue samples of the URSA patients resulted in strong STAT3 activation, which is consistent with the results of a previous study [33].

**Fig 4:**
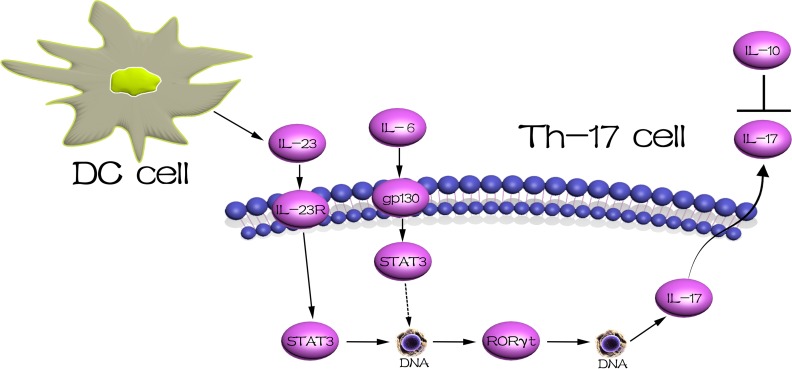
Model of the regulatory networks of signal transducers and activators of transcription 3(STAT3) and related cytokines. Interleukin (IL)-23 binds with its receptor IL-23R, and IL-6 binds with its membrane receptor, which could activate the STAT3 protein and promote the transcription of retinoic acid-related orphan receptor gammaT (RORγt). This cascade then promotes the secretion of IL-17, and IL-10 could inhibit the secretion and function of IL-17.

In conclusion, our results describe the immunoregulatory events that modulate maternal-fetal immunotolerance. Based on our data, we hypothesize that the STAT3 signaling pathway is activated at the maternal-fetal interface in response to high levels of IL-23 and IL-17, and that this pathway likely plays a key role in the pathogenesis of URSA (Fig 4). However, further research is necessary to evaluate whether IL-23 can enhance IL-23R expression through the STAT3 signaling pathway, and to determine whether the use of specific inhibitors of IL-23 may comprise a new strategy for the treatment of URSA. In addition, studies are needed to verify whether STAT3 is activated in response to the binding of the above-mentioned cytokines to their specific receptors, and whether this activation results in the stimulation of RORγt transcription and subsequently to the inflammatory reactions that lead to abortion.

## Supporting Information

S1 Fig A picture of the decidua. (TIF)

S2 Fig A picture of the villus. (TIF)

S3 Fig Western blot images. Western blot results showing the levels of STAT3, phosphorylated STAT3, and IL-17 receptor protein in decidual tissue samples from women in the unexplained recurrent spontaneous abortion(URSA) group and the control group.
(RAR)

S1 Table Cytokine expression in the URSA patients and normal controls. Enzyme-linked immunosorbent assay data of the IL-6, IL-10, IL-17, and IL-23 levels in peripheral blood and decidual tissue samples from women in the unexplained recurrent spontaneous abortion(URSA) group and the control group.
(XLSX)
