# Supplementary figures and images for: Differences in Cytokine Expression and STAT3 Activation between Healthy Controls and Patients of Unexplained Recurrent Spontaneous Abortion (URSA) during Early Pregnancy
Source: PLoS One. 2016 Sep 22;11(9):e0163252. doi: 10.1371/journal.pone.0163252 (PMC5033573; doi:10.1371/journal.pone.0163252)

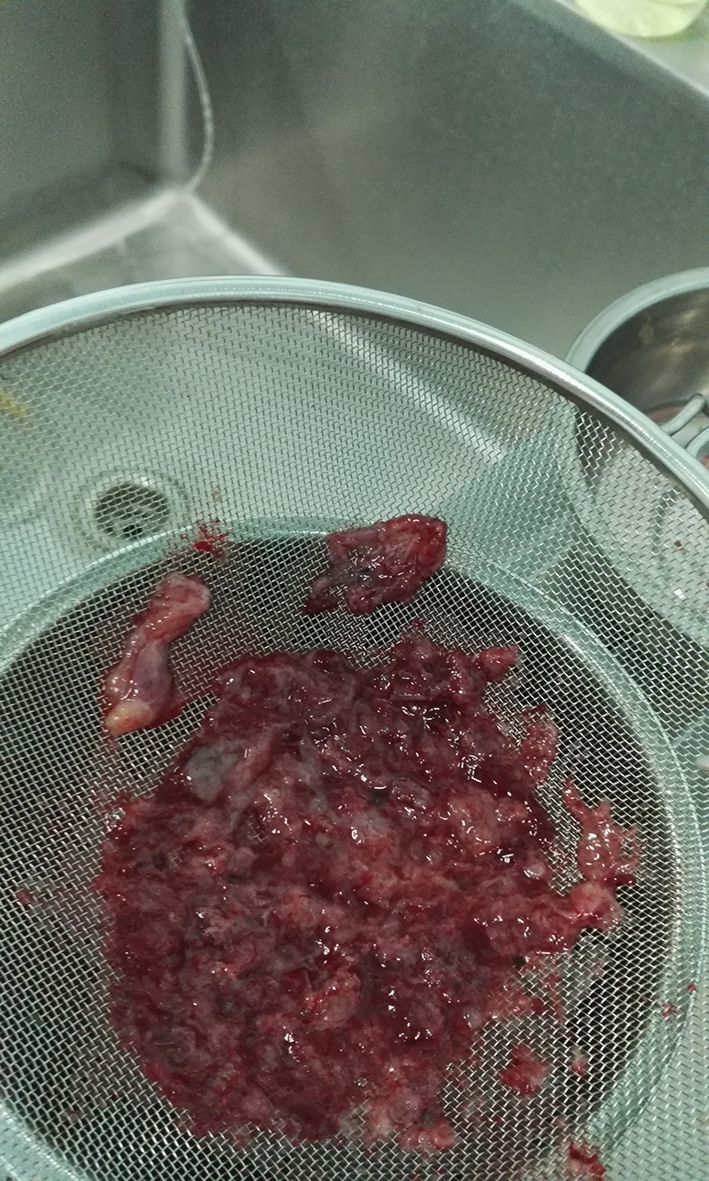

Supplement: S1 Fig — (TIF) [file pone.0163252.s001.tif]

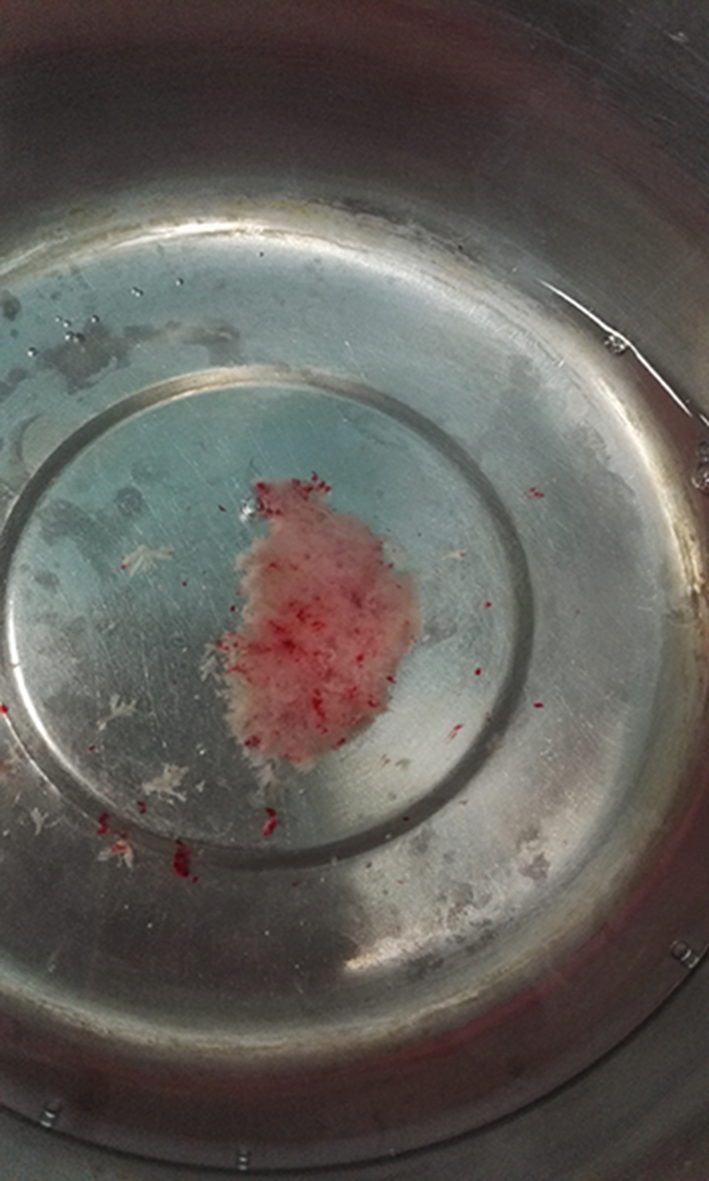

Supplement: S2 Fig — (TIF) [file pone.0163252.s002.tif]
